# Weather fluctuation can override the effects of integrated nutrient management on fungal disease incidence in the rice fields in Taiwan

**DOI:** 10.1038/s41598-022-08139-7

**Published:** 2022-03-11

**Authors:** Ming-Chih Chiu, Chi-Ling Chen, Chun-Wei Chen, Hsing-Juh Lin

**Affiliations:** 1 Department of Life Sciences and Innovation and Development Center of Sustainable Agriculture, National Chung Hsing University, Taichung City, 40227 Taiwan; 2 Department of Entomology, National Chung Hsing University, Taichung City, 40227 Taiwan; 3 Taiwan Agricultural Research Institute, Council of Agriculture, Executive Yuan, Taichung City, 41362 Taiwan

**Keywords:** Ecology, Agroecology

## Abstract

Both weather fluctuation and farming system influence the epidemiology of crop diseases. However, short-term experiments are difficult to mechanistically extrapolate into long-term ecological responses. Using a mechanistic model with Bayesian inference, long-term data spanning 10 years were used to construct relationships among weather fluctuation (temperature, relative humidity, wind, and rainfall), farming system (conventional and low-external-input farming), and crop disease in experimental rice fields in Taiwan. Conventional and low-external-input farming had similar influences on the disease incidence of rice blast. Temperature had a positive influence on the disease incidence only under high relative humidity. Rainfall positively affected the disease incidence until an optimum level of rainfall. Low-external-input farming, with a lower application of fertilizers and other sustainable nutrient management, achieved similar effects on the disease incidence to those achieved by conventional farming. This suggests that weather fluctuation may override the effect of the farming systems on fungal disease incidence in rice fields.

## Introduction

Understanding the roles of weather fluctuation and farming systems in plant disease epidemics can help elucidate the interactions between plants and their hosts under field conditions^1^. The altered prevalence of plant diseases due to climate variability has highlighted the challenges in predicting the incidence and severity of major diseases in important crops (e.g., rice) based on field conditions^2^. Disease control can be achieved via agronomic management by maintaining biodiversity and improving soil health through nitrogen-fixing and cover crops, intercropping, manure and compost applications, and reduced tillage^3–5^. Therefore, management of plant diseases under climate aspects and farming systems are crucial to secure and improve our current crop production.

Weather fluctuation impacts epidemiological features of plant pathogens and the incidence and severity of plant diseases^6,7^. For example, an increase in temperature leads to an increased risk of disease and the spread of pathogens to new geographic areas in many pathosystems^6,8^. Water stress could decrease the resistance of plant hosts to pathogen infections and enhance disease development^9^. The development of plant diseases occurs within a range of suitable environmental conditions, e.g., accelerated fungal infections by increased precipitation occurring not beyond optimal levels of precipitation^10^. In addition, disease development is associated with the combined effects of climatic factors, e.g., high establishment of blast pathogen occurring at moderate temperatures (e.g., 22 °C) with high humidity (e.g., > 90% RH) during nights^11^. The exploration of plant-pathogen interactions is complicated by weather fluctuation because environmental conditions can influence the biological traits of pathogens, such as the production and germination of propagules and pathogen growth rates^12^.

Farming systems change the field conditions, by influencing the development of pathogens and/or the vulnerability of crop plants to diseases^3^. Sustainable agriculture relies on management that considers ecological processes, biodiversity, and recycling to ensure plant health and crop performance, whereas conventional agriculture implements a significant amount of synthetic chemicals to maintain crop plants. Compared to conventional agriculture, sustainable agriculture decreases or even prohibits the use of synthetic fertilizers and pesticides. Disease resistance and tolerance of crop plants are potential factors that can reduce diseases, and the effects of these factors on diseases can be influenced by the level of nutrients applied^13^ but are primarily modulated by crop genetics. For example, nitrogen increases the crop susceptibility, triggered by resistance genes, to the blast disease caused by the fungus *Magnaporthe oryzae* in rice and wheat^14^. According to Reddy, et al.^15^, some bacterial leaf blight caused by *Xanthomonas oryzae* pv. *oryzae* was observed in rice under high nitrogen fertilization (e.g., 180 kg N ha^−1^), and the rapid expansion of rice sheath blight (caused by *Rhizoctonia solani*) was observed in an area with a nitrogen supply (from zero to 40 kg N ha^−1^) in tropical Asia^16^. Despite the generally higher suppression of plant diseases in sustainable agriculture when compared with conventional agriculture^3^, exceptions occur in particular pathosystems and under certain local conditions, e.g., elevated levels of foliar pathogens and aphid-borne viruses in organic wheat fields^17^.

With long-term information (e.g., more than 10 years), few studies comprehensively address the mechanistic linkages between climate, farming systems, and crop diseases. Results obtained in short-term experiments are generally difficult to extrapolate to predict the long-term responses of crop plants to weather fluctuation^18^. Mechanistic inferences with long-term information, which enables more robust results with which to consider the impacts of weather fluctuation on diseases in paddy fields, are still scarce, although rice is one of the most important crops in the world, especially in Asia. The symptoms can be lesions on all parts of the plant (e.g., leaves, leaf collars, necks, panicles, pedicels, and seeds). The disease spreads by windborne spores, by water, and by contact with infected plant materials.

To fill this knowledge gap, we applied a mechanistic model with Bayesian inference to quantify the nonlinear effects of climate factors on a major rice disease (rice blast caused by *Pyricularia oryzae*) in experimental rice fields under conventional and low-external-input farming systems based on 10 years of data from 2006 to 2016. Our model explicates important processes in which key weather factors (e.g., rainfall and temperature) have actions, which have been intergraded in previous models^19–21^. Furthermore, we considered the influences of farming systems into the model. In mathematical statistics, Bayesian inference is a method of statistical inference used to estimate posterior distributions of model parameters. Herein in the rice-growing areas being investigated, we tested a hypothesis that farming system and weather fluctuation have joint influences on disease development. In this study, the farming systems were considered as integrated nutrient management with the application of fertilizers and other sustainable nutrient inputs.

## Results

### Weather fluctuation

**Figure 1:**
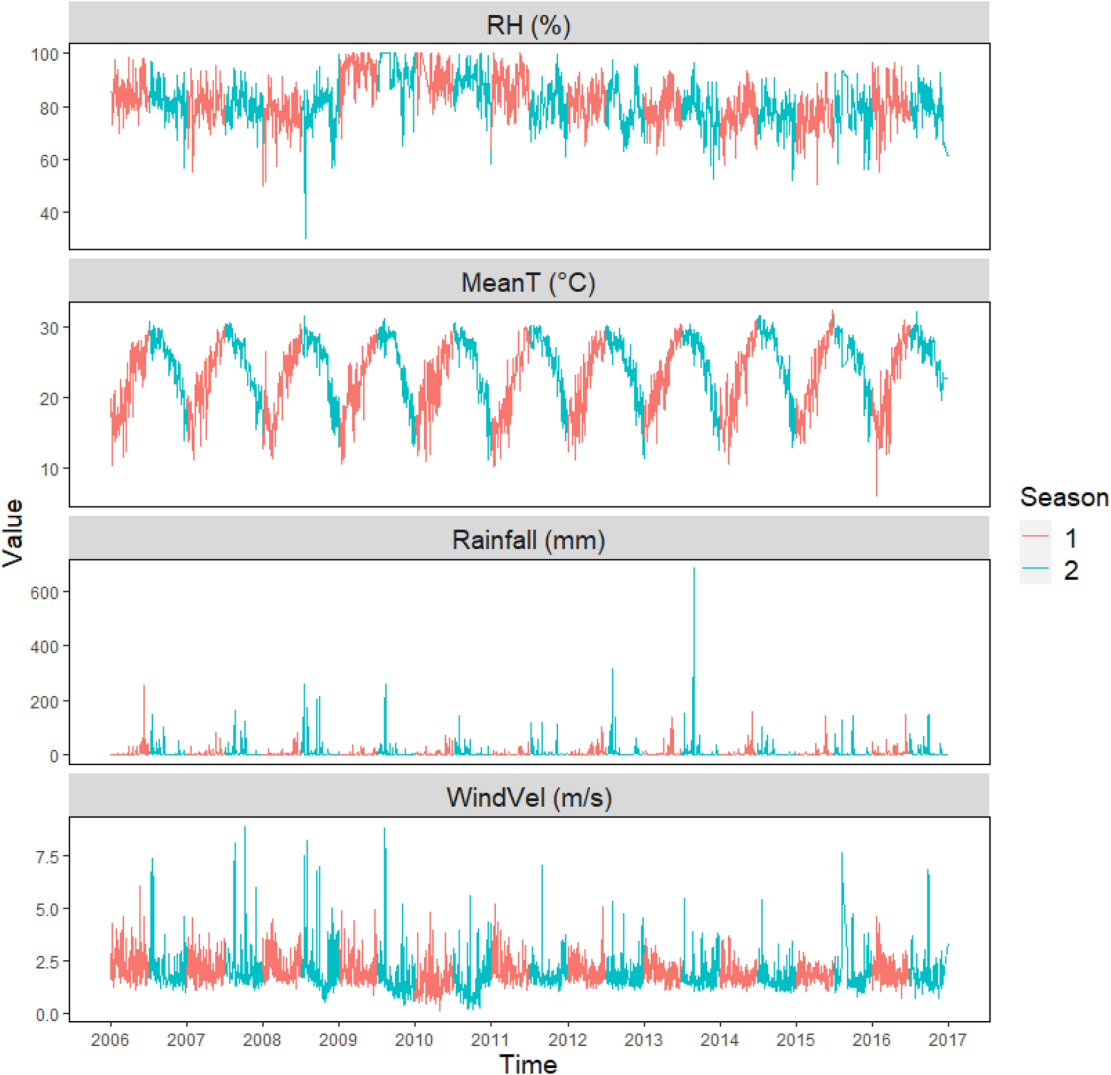
Daily relative humidity (RH), temperature (MeanT), rainfall, and wind velocity (WindVel) from 2006 to 2016 in central Taiwan. Growing seasons 1 and 2 occur in the first and second halves of the year, respectively.

The daily temperature and rainfall showed more obvious seasonal changes than the daily relative humidity and wind velocity. In addition, the other climatic factors (i.e., rainfall, relative humidity, and wind velocity) showed higher annual changes than that of daily temperature (Fig. 1). The daily temperature showed seasonal changes that ranged from 6 °C in the winter (December to February) to 33 °C in the summer (June to August). Daily rainfall exhibited a similar seasonal pattern. Daily rainfall was higher during the wet season (May to October) and lower during the dry season (November to April). The annual maximum daily rainfall typically ranged from 119 to 307 mm, but extreme daily rainfall occurred in 2013 (682 mm; Fig. 1). However, daily relative humidity did not show consistent seasonal changes. The annual maximum daily relative humidity (93 to 100%) occurred in all seasons. From 2009 to 2011, high daily relative humidity (more than 90%) occurred more frequently (89 to 261 days per year) when compared with other years (7 to 54 days per year). Daily wind velocity was usually higher in the summer compared with other seasons (Fig. 1).

### Disease incidence

The incidence of rice blast exhibited seasonal changes, and except in 2013, the incidence was higher in the first half of the year than the second (growing season average: 100 and 15% at most in the first and second growing seasons, respectively; Fig. S2). Similarly, rice blast had the highest observed values of disease incidence ($RAUDPC$; relative area under the disease progress curve) in the first half of the year when compared with the second half of the year, except in the year 2013 (Fig. 3). Rice blast had the highest peaks of incidence and $RAUDPC$ in the first half of the year in 2010 and 2011 (Fig. S2 and Fig. 3).

### Bayesian modeling

**Figure 2:**
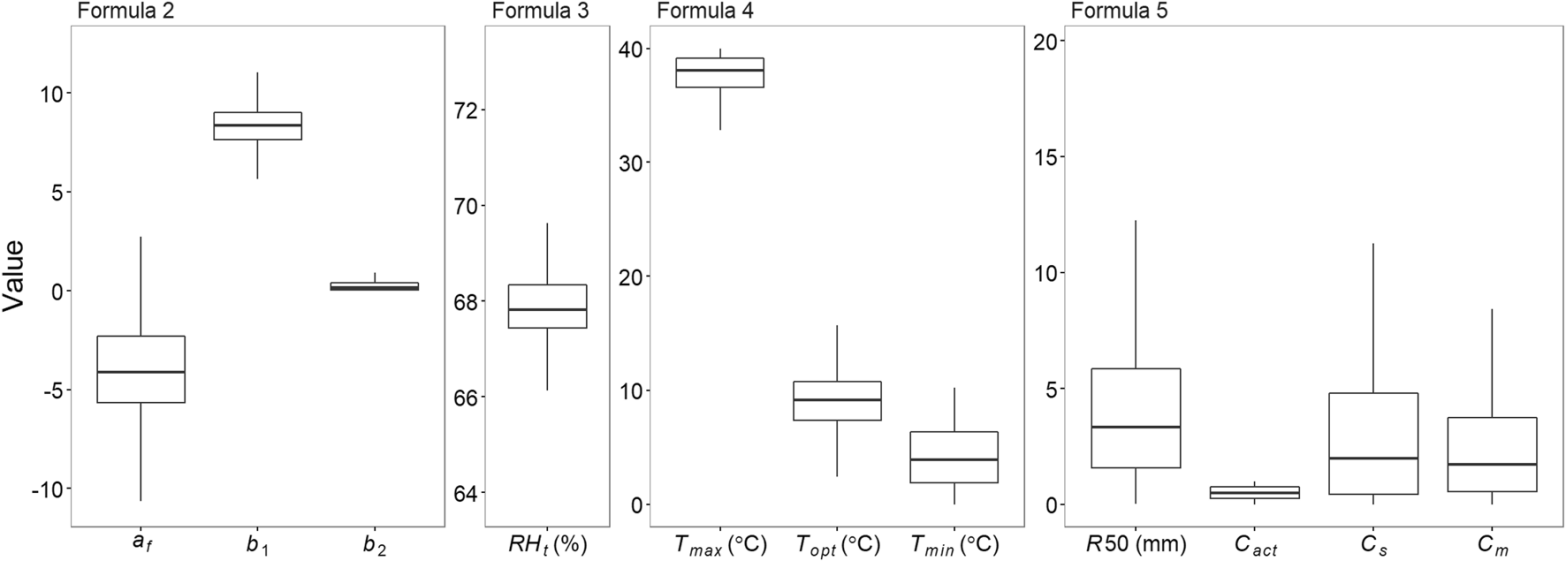
Bayesian estimation of the model parameters and their posterior distributions in the best model without considering the difference between conventional (CF) and low-external-input farming (LF) (based on the same value of the constant baseline ${a}_{f}$ for different farming systems in Formula (2)) among the two competing models. The other model considers the difference based on two different values of constant baseline ${a}_{f}$.

**Figure 3:**
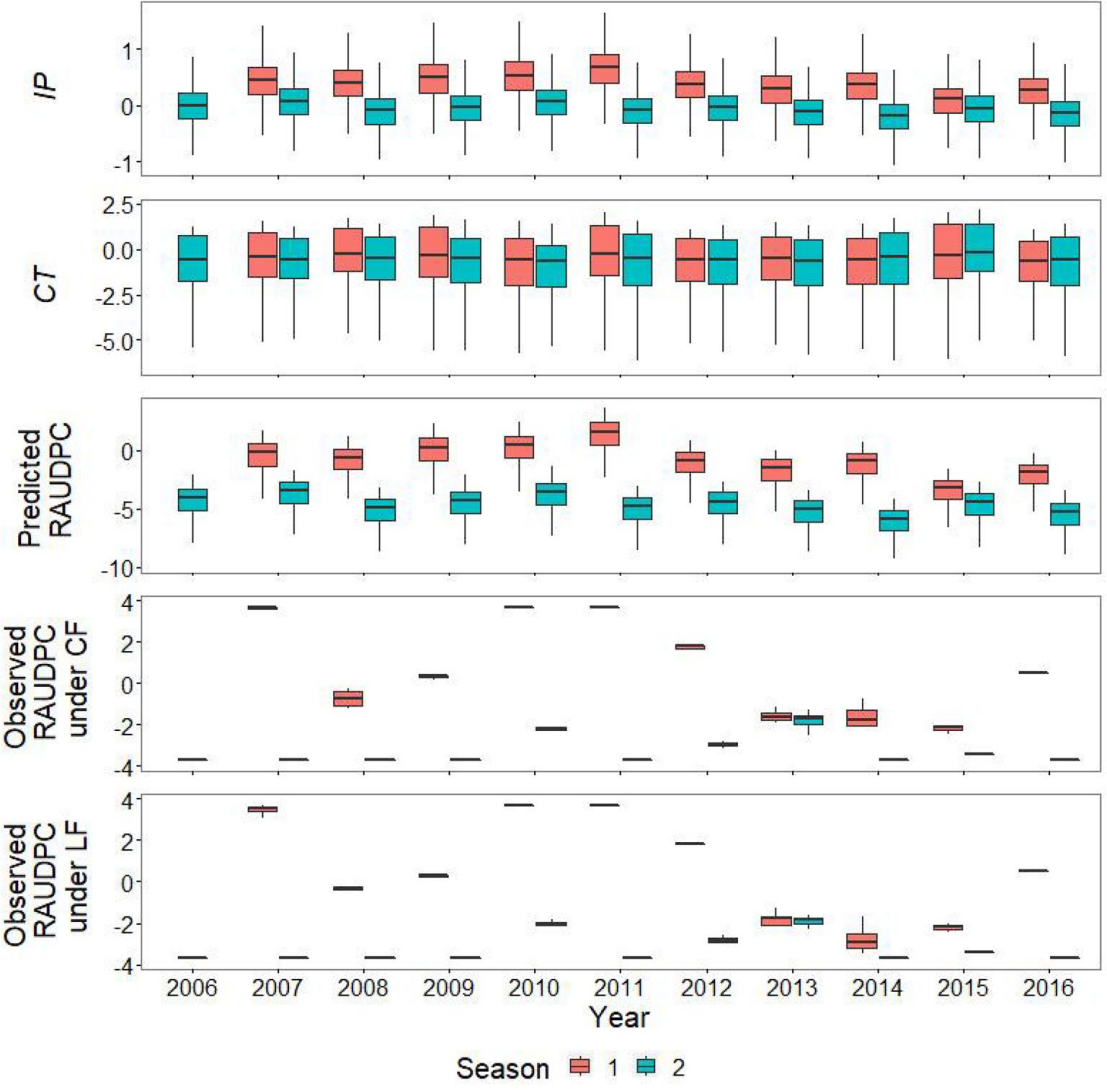
The progress rate of the development of primary inoculum ($IP$), the net catchment of the airborne spores by rainfall and wind onto the leaf area ($CT$; when subtracting the detachment of spores by rainfall from the host tissue) , and the predicted value of the relative area under the disease progress curve ($RAUDPC$) for rice blast and the observed $RAUDPC$ for rice blast under conventional and low-external-input farming (CF and LF, respectively) from 2006 to 2016 in the rice fields of central Taiwan. Growing seasons 1 and 2 occur in the first and second halves of the year, respectively. All values of each variable were logit-transformed.

Based on the lowest WAIC (widely applicable information criterion; 568.2 and 571.2, respectively), the best model without considering the difference between the two farming systems (i.e., high chemical fertilizer input, CF, and low-external-input farming with low fertilizer input, LF) had a good fit (*R*^2^ = 0.75; the *R*^2^ derived from the model residuals). The model performance was similar to the other model with this consideration (*R*^2^ = 0.75). In addition, the best model (i.e., without the consideration) had a higher value of ELPD (expected log predictive density) and the predictive ability than the other model (ELPD = -284.1 and -285.6, respectively). The influences of the CF and LF systems on the incidence of rice disease were similar (Fig. 3 and Fig. S2). The best model indicated that weather fluctuation (i.e., temperature and rainfall) had nonlinear effects on rice disease (Figs. 2, 3, Fig. S2, and Formulas (2) to (4)). However, decreased temperature and/or rainfall usually enhanced the $RAUDPC$ of rice blasts during our study period (Figs. 1 and 3). High values of relative humidity and wind velocity helped disease development (Figs. 1 and 3).

## Discussion

This study assessed the influence of both weather fluctuation and farming systems on crop disease incidence and supports the hypothesis that weather fluctuation could alter the disease development. El Niño–Southern Oscillation (ENSO) can be linked to climate variability at seasonal and interannual time scales, especially for rainfall in Taiwan^22^. However, a more dramatic change in rainfall, compared with other weather factors, seemed not associated with ENSO in our study area (Fig. 1 and Fig. S1). Our results suggest that experimental assessment of the long-term efficacy of agricultural management is necessary before being put into practice for helping disease control in rice fields across large areas. In the subsequent discussion, we evaluate whether the relationships could provide possible mechanisms explaining the variation in crop disease incidence. In addition, some biotic/abiotic factors (e.g., rice variety) that reduced the ability of climate variables and farming systems to be used as predictors of disease state and suggestions for future studies on disease issues are discussed below.

### Effects of weather fluctuation

Temperature and rainfall were both crucial to crop disease and rice production in our paddy fields. Simultaneous changes in rainfall and temperature do not cause the same (either favorable or adverse) effects on pathogens^23^. The development of crop disease occurs within a range of suitable environmental conditions^24,25^. For example, the germination of fungal spores (*P. oryzae*), which results in rice blast and their infection, increases with temperature until optimal temperatures are reached^26^. Rice disease development is expected to have optimal temperature and rainfall conditions, but the two climatic factors might be close to or beyond the optimum in low-latitude Taiwan, which experiences monsoon seasons. Therefore, we did not detect a nonlinear relationship but we did detect a negative effect of temperature rises on the incidence of rice blast in the second half of the year. In addition, the temporary differences of climate caused the seasonal incidence of rice blast in our study area. In other study, the *M. oryzae* fungus can survive under large thermal oscillations but negative temperatures reduce its sporulation rate in the wheat residues^27^.

An increased amount of rainfall could also have negative effects on disease transmission. Rainfall could accelerate fungal infections by dispersing fungal spores via rain splash or by adhering them to rice leaves; similar phenomena have been observed in a variety of fungi e.g.,^10,28,29^. Crop epidemics can efficiently spread because infectious fungi can propagate rapidly across neighboring plants. We verified that rainfall is an important factor for the disease incidence, but it did not have higher relative importance (Fig. 3). El Jarroudi, et al.^30^ suggested that rainfall is important only for the initiation of infection (≤ 1 mm) in wheat because of its role in the process of adhesion. According to Penet, et al.^31^, dispersal directly from a source is the main mode of spore dispersal by rain splashes and that indirect dispersal via resplash has a smaller impact but can still contribute to spore contamination to a certain extent. For diseases caused by different pathogens (e.g., bacterial leaf blight) in rice fields, rainfall could result in an opposite effect because the guttation fluid on the infected leaf, which is inoculated by the bacterial population, can be washed off^32^. Therefore, high rainfall did not show different effects on pathogen infections and might even prevent disease development, especially for rice blasts, over our study duration.

Conversely, a single environmental factor favorable to diseases might not facilitate their development, and the interaction and combined effects of climatic factors should be considered. In some years, the moderate temperature under moderate rainfall could result in a higher incidence of rice blast in our study area. The inference can be also applied to the seasonal weather conditions and higher incidence of rice blast in the first half of the year. Disease development does not rely on a single environmental factor, but the combined effects of different climatic factors (e.g., air temperature and rainfall in our study) can influence pathogen infection. In addition to ambient temperature, leaf wetness, which is determined by multiple meteorological variables (e.g., air relative humidity and rain occurrence), usually plays a fundamental role in the development of infection from fungal pathogens^2,21^. Temperatures close to the optimal temperature for infection allowed a shorter wet period for fungal germination^30^. In addition, blast pathogen can have high establishment when peak spore release occurs at moderate temperatures (e.g., 22 °C) with high humidity (e.g., > 90% RH) during nights^11^. In the current study, relative humidity might not have been the limiting factor for the wetness requirements of *R. solani* to cause sheath blight^26,33^ because the moisture in the air is already high in Taiwan.

### Effects of farming system

Conventional and low-external-input farming systems had no difference in depressing the rice disease in our fields. In rice and other crop fields, agronomic management can influence the vulnerability or the resistance to crop diseases^34–36^ and could be effective ways to mitigate and control epidemics^37^. In general, conventional farming utilizes chemical fertilizers, which may cause a high nitrogen content in crop plants and boost pathogen infections^13,38,39^. Organic inputs could reduce the outbreak of diseases (e.g., rice blast and sheath blight) more than NPK (Nitrogen, Phosphorous, and Potassium) fertilizers in rice fields^35^. Because the best model was selected without considering the difference between the two farming systems, we suggest that the integrated nutrient management (e.g., chemical fertilizer and compost applications) had no effect on the development of crop disease in the rice fields. Thus, climate variability might override the depression effect of the low-external-input farming system on rice diseases. Other study found a positive association between the severity of wheat blast (caused by fungus *M. oryzae*) and the amount of nitrogen fertilization in Brazil^40^.

### Conclusions and overview

Weather fluctuation has a critical role in our systems, which might override the acting of the farming systems in managing the major rice diseases. Weather fluctuation has threatened rice production and continues to influence its socioeconomic value^41^. For example, the impacts of climatic factors on rice production have influenced the net income of the farmers in Malaysia^42^. Integrating the pathogens of crop plants into the consideration of climate aspects can help resolve the food security debate^43^. Any effort to manage crop diseases will have noticeably positive effects on the enhancement of global food security^44^. A mechanistic understanding of disease development under climate variability could promote realistic predictions in agricultural ecosystems^45^. Thus, our study could provide research contributions for Taiwan or the world. Given the seasonal incidence of rice blast, we suggest that the management efforts, i.e., for controlling rice blast disease, should be applied more to the first than second half of the year. In the future, winter warming is predicted to occur in Taiwan^46^, which could allow the approaching towards the temperature optima for the development of rice diseases (e.g., rice blast). The inference raises the concerns of food sustainability and leads our management considerations in the future.

## Materials and methods

### Plant material

Rice (*Oryza sativa* L.) plants used for the experiment were from the collection of Taiwan Agricultural Research Institute. The rice variety (Tainan No. 11) used in this study has enhanced resistance to rice blast. The use of plant materials complies with international, national, and/or institutional guidelines and legislation.

### Field area

This study was carried out in experimental rice fields under low-external-input and conventional farming in central Taiwan (23.5859 N, 120.4083 E; 8.0 ha). The annual average temperature ranged from 23 to 25 °C, the annual average relative humidity ranged from 75 to 92%, and the annual rainfall ranged from 1020 to 2873 mm year^−1^ (average data between 2006 and 2016 measured at a nearby weather station; Fig. 1). The experimental paddy plots were defined by considering the typical dimensions of the agricultural fields in Taiwan (0.5 to 1.0 ha). A long-term experiment was conducted from 2006 to 2016 to study the effects of different agronomic management on biodiversity, productivity, and environment, including traceability system, soil fertility, nitrogen leaching, production costs, disease incidence and severity, the abundance of pest and beneficial insects, and weed dynamics.

The treatments consisted of conventional farming with high chemical fertilizer input (CF) and low-external-input farming with low fertilizer input (LF). In the CF farming, we followed the fertilizer recommendations that are constructed to meet the nutrient requirements of the crop. In the LF farming, the chemical fertilizers were largely reduced compared to the recommendation (see next paragraph for the details). The experiment was conducted as a randomized complete block design (RCBD) with four replicates. In agricultural experiments, the RCBD is a standard procedure by grouping experimental units into blocks. For example, the design can control variation in the experiments by considering spatial influences and adjusting the effects of target factors in fields. Each experimental unit consisted of a 0.58 ± 0.16 ha of the area of the field. Additional nutrient management in the LF system includes (1) nitrogen-fixing and cover crops, (2) manure and compost applications, (3) plant and soil nutrient analyses for adjusting fertilization, and (4) reduced tillage. Soil-available potassium gradually decreased during the 10-year study period in the area of the LF system. Over the study period, the LF system achieved the similar level of crop production as that of the CF system (Fig. S1).

In our study area, there were two growing seasons within a year: one in the first half of the year (from February to June) and one in the second half (from August to December). The ground fertilizers were applied before rice seedlings were transplanted, followed by additional fertilizations during the tillering and boosting stages. The total amount of fertilizers used for the CF system included 140–180 and 120–140 kg N ha^−1^, 70–72 and 60 kg P_2_O_5_ ha^−1^, and 85 and 60 kg K_2_O ha^−1^ for the first and second seasons, respectively. For the LF system, 100 and 80 kg N ha^−1^, 30 and 30 kg P_2_O_5_ ha^−1^, and 30 and 30 kg K_2_O ha^−1^ were applied in the first and second seasons, respectively. The larger amount of fertilizers for the first season was due to its longer duration. For each rice growing season, fungicides were applied to both farming systems once during the boosting stage. During the fungicide application, a 10% mixture of Cartap plus Probenazole or 6% probenazole for rice blast (both 30 kg ha^−1^) and 1.5% Furametpyr for sheath blight (20 kg ha^−1^) were used.

### Rice disease monitoring

The major rice disease (rice blast; Fig. S2) was monitored biweekly in the CF and LF systems over the two growing seasons per year, with each growing season including (in chronological order) the tillering, flowering, and maturing stages. There was a total of 123 occasions during our study. The plants were disease free when planted out. When the lesion of the rice blast began to appear in the fields from the tillering stage to the maturing stage, the effects of the two treatments (CF and LF systems) in the paddy fields on the disease incidence of rice blast (caused by *Pyricularia oryzae*) were investigated. For each plot (or experimental unit), the incidence of rice disease was randomly examined at 5 points and for 25 plexuses (i.e., each derived from one primary tiller) per point. The disease incidence was quantified as the percentage of infected plexuses that were determined based on the presence of infected leaves.

The area under the disease progress curve (AUDPC) was used to quantify disease incidences over time, and the relative AUDPC ($RAUDPC$) was used because of unequal sampling duration in the growing seasons during our study period. For each plot (or experimental unit), we used the $RAUDPC$ to summarize the incidences of disease during each growing season as follows:

1 $$RAUDPC=\frac{\sum_{i=1}^{n-1}\frac{{y}_{i}+{y}_{i+1}}{2}\times \left({t}_{i+1}-{t}_{i}\right)}{100 \times \left({t}_{n}-{t}_{1}\right)},$$

where ${y}_{i}$ and ${t}_{i}$ are the disease incidence (%) and time (day) at the $i$th observation, respectively, and $n$ is the total number of observations.

### Bayesian modeling

We built a mechanistic model that was applied to assess the interplay within a network of relationships among weather fluctuation, farming system, and disease incidence in the paddy fields. The model describes how (1) temperature and relative humidity together influence the development of primary inoculum, (2) rainfall detaches the fungal spores on the host tissues, and (3) rainfall and wind catch the airborne spores onto the leaf area. These environmental processes determine the disease incidence in the model. In addition, this model considers that farming systems can suppress or accelerate disease incidence. By fitting our model to the observed incidence, Bayesian inference was used as the parameter estimation technique for the models. In addition, we tested the alternative mechanistic hypotheses based on a model-selection criterion and cross vaidation (see subsequent paragraphs).

With a linearity assumption, the incidences of disease $RAUDPC$ were modeled as an inverse-logit function of the progress rate of the development of primary inoculum ($IP$ with values between 0 and 1) and the net catchment of the airborne spores by rainfall and wind ($CT$ with values between 0 and 1; when subtracting the detachment of spores by rainfall from the host tissue) as follows:

2 $$RAUDPC=invLogit\left({a}_{f}+{b}_{1}\bullet logit\left(avg\_IP\right)+{b}_{2}\bullet logit\left(avg\_IP\bullet avg\_CT\right)\right),$$

where ${a}_{f}$, ${b}_{1}$, and ${b}_{2}$ describe the constant baseline for different farming systems ($f$ = the CF or LF system), the direct effect size of $avg\_IP$, and the mediating effect size of $avg\_CT$ through $IP$, respectively. The two parameters ($avg\_IP$ and $avg\_CT$) are averaged $IP$ and $CT$ during the growing season, respectively (see below for details). The effect sizes ${b}_{1}$ and ${b}_{2}$ have values more than zero. The constant baseline allows the management-specific acting in the model when they can influence the disease incidence.

The process rate $IP$ was simulated as a function of the temperature response ($f\left(T\right)$ with values between 0 and 1) and hourly air relative humidity $(RH,$ %) as follows^20^:

3 $$IP= \left\{\begin{array}{ll}0& \mathrm{if}\, RH<{RH}_{t} \, or \,  LW= \mathrm{false} \\ f\left(T\right)& \mathrm{elsewhere}\end{array}\right.,$$

where ${RH}_{t}$ the threshold of air relative humidity and $LW$ is the leaf wetness. Here, we used the RH condition because of the data availability. The relative humidity if lower than the threshold inhibits inoculum development^47^. Otherwise, the development of the primary inoculum is promoted, relying on the change in temperature. Herein, $f\left(T\right)$ describes the development of the primary inoculum under temperature change^19^:

4 $$f\left(T\right)=\left(\frac{{T}_{max}-T}{{T}_{max}-{T}_{opt}}\right)\cdot {\left(\frac{T-{T}_{min}}{{T}_{opt}-{T}_{min}}\right)}^{\frac{{T}_{opt}-{T}_{min}}{{T}_{max}-{T}_{opt}}},$$

where $T$ is the hourly air temperature (°C). We denoted the ${T}_{min}$, ${T}_{max}$, and ${T}_{opt}$ pathogen-specific cardinal temperatures (minimum, maximum, and optimal temperatures, respectively) for inoculum development. To meet valid values of $f\left(T\right)$, the minimum and maximum temperatures are considered as out of the two ends of the measured range. The temperature response function $f\left(T\right)$ is also associated with the sporulation efficiency^20^.

The net catchment of the airborne spores ($CT$) was simulated as a nonlinear function of rainfall and wind velocity as follows^20,48^:

5 $$CT = \underbrace {{{0.9}^{\frac{R}{{{C_{act}}}}}}}_{{C_R}} \cdot \underbrace {\frac{1}{{1 + {\text{exp}}( - {C_s}\left( {W - {C_m}} \right))}}}_{{C_W}} \cdot \left( {1 - \underbrace {\frac{R}{{R50 + R}}}_{{D_R}}} \right),$$

where ${D}_{R}$ (between 0 and 1) describes the direct detachment of spores by rainfall from the host tissue, in which $R$ is the daily rainfall (mm) and $R50$ is the rainfall able to provoke detachment of 50% of the spores. ${C}_{R}$ and ${C}_{W}$ (both between 0 and 1) describe how rainfall and wind allow airborne spores caught on the leaf area, respectively. ${C}_{act}$ (between 0 and 1) is the actual portion of spores caught at a rainfall of 1 mm. $W$ is the daily wind velocity (m s^−1^), and ${C}_{s}$ and ${C}_{m}$ (both > 0) are the steepness and midpoint parameters to control the portion of spores caught by the wind, respectively.

The Bayesian framework ‘Stan’^49^ and its R interface ‘RStan’^50^ were used to construct and fit the models. There were two competing models: either considering the difference between the CF and LF systems by not fixed to the same values of the constant baseline ${a}_{f}$ in Formula (2) or not. For each model, four Markov Chain Monte Carlo (MCMC) chains (for numerical approximations of Bayesian inference) ran, each with 5,000 iterations, and the first half of the iterations of each chain were discarded as burn-in. The R-hat statistic of each parameter approaches a value of 1, indicating model convergence. With a total of 2,000 samples, collected as one sample for every 5 iterations for each chain, the model parameters and their posterior distribution were estimated. To compare the two competing models, we calculated the widely applicable information criterion (WAIC) using the R package ‘loo’^51^. The best model was determined based on the lowest WAIC. By using the same R package, we also performed the approximate leave-one-out cross-validation (LOO-CV) to estimate the predictive ability of the two Bayesian models. Here, we used the expected log predictive density (ELPD) to be the predictive performance.

### Compliance with ethical standards

The authors declare that they have no conflict of interest. This article does not contain any studies involving animals performed by any of the authors. This article does not contain any studies involving human participants performed by any of the authors.

## Supplementary Information

Supplementary Figures.
