# Supplementary material for: Weather fluctuation can override the effects of integrated nutrient management on fungal disease incidence in the rice fields in Taiwan
Source: Sci Rep. 2022 Mar 11;12:4273. doi: 10.1038/s41598-022-08139-7 (PMC8917239; doi:10.1038/s41598-022-08139-7)
Supplement: Supplementary file 1 — Supplementary Figures. [file 41598_2022_8139_MOESM1_ESM.docx]

**Appendix**


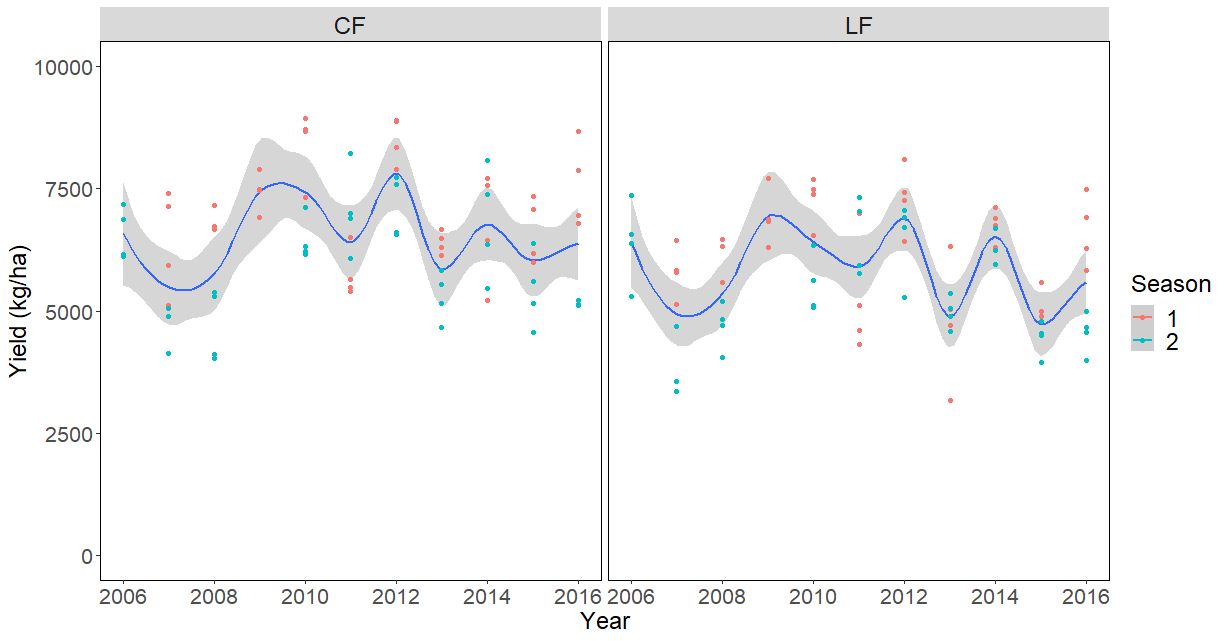


**Fig. S1.** Crop production under conventional and low-external-input farming (CF and LF, respectively) from 2006 to 2016 in the experimental paddy fields of central Taiwan. Each point indicates observed yield. Growing seasons 1 and 2 occur in the first and second half of the year, respectively. The blue line and gray area indicate trajectory and 95% confidence interval, respectively, based on LOWESS (locally weighted scatterplot smoothing).


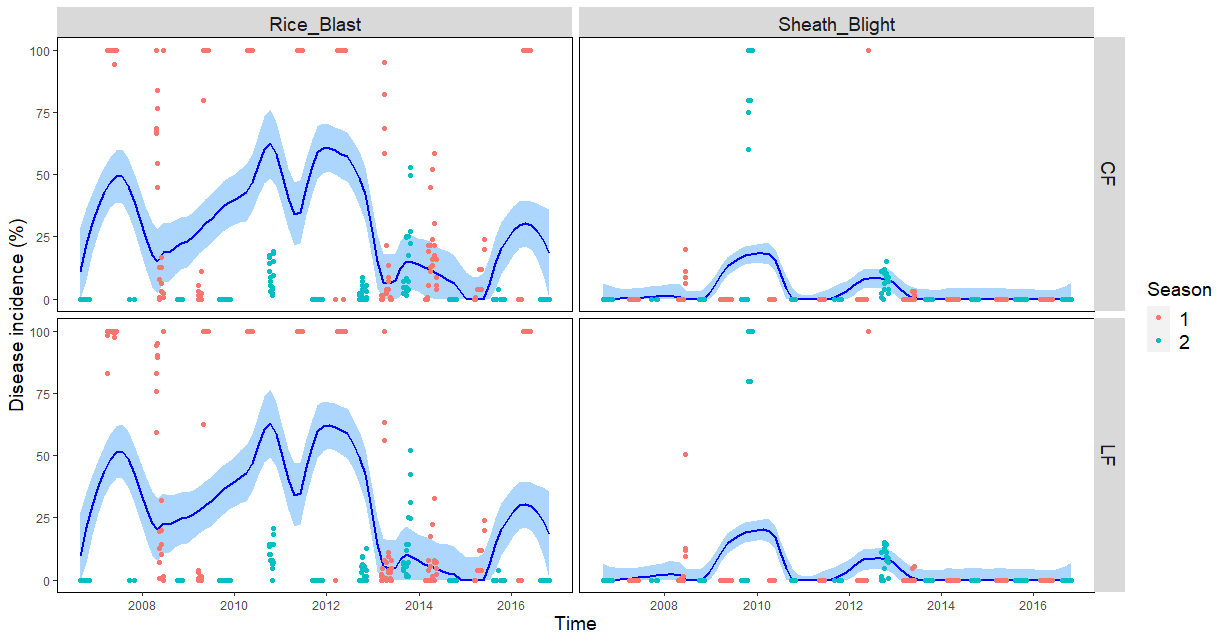


**Fig. S2.** Incidences of rice blast and sheath blight under conventional and low-external-input farming (CF and LF, respectively) from 2006 to 2016 in paddy fields of central Taiwan. Each point indicates observed yield. Growing seasons 1 and 2 occur in the first and second half of the year, respectively. There were multiple observations during each growing season. Blue line and area indicate trajectory and 95% confidence interval, respectively, based on LOWESS (locally weighted scatterplot smoothing).


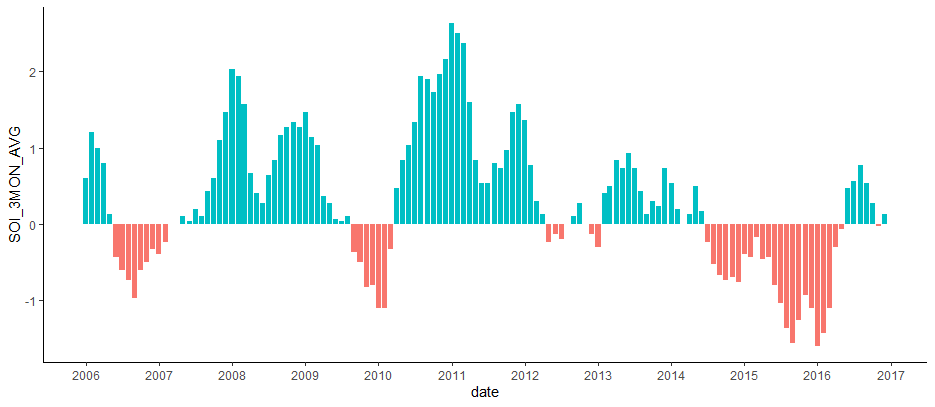


**Fig. S3.** Three month moving average of the Southern Oscillation Index (SOI; normalized pressure difference between Tahiti and Darwin) from 2006 to 2016.
